# Phytochemical Characterization and Biological Activity of Two *Anacardiaceae* Species from Guinea-Bissau

**DOI:** 10.3390/plants14010008

**Published:** 2024-12-24

**Authors:** Quintino Malú, Maryam Malmir, Gonçalo Infante Caldeira, Sofia Encarnação, Katelene Lima, Luís Catarino, Beatriz Silva Lima, João Rocha, Olga Silva

**Affiliations:** 1 Research Institute for Medicines (iMed.ULisboa), Faculty of Pharmacy, Universidade de Lisboa, 1649-003 Lisbon, Portugal; quintinomalu@edu.ulisboa.pt (Q.M.); m.malmir@edu.ulisboa.pt (M.M.); goncalo.caldeira@edu.ulisboa.pt (G.I.C.); sofia.encarnacao@edu.ulisboa.pt (S.E.); k.lima@edu.ulisboa.pt (K.L.); mblima@ff.ulisboa.pt (B.S.L.); joao.rocha@edu.ulisboa.pt (J.R.); 2 Centre for Ecology, Evolution and Environmental Changes (cE3c) & CHANGE-Global Change and Sustainability Institute, Faculty of Sciences, Universidade de Lisboa, 1749-016 Lisboa, Portugal; lmcatarino@fc.ul.pt

**Keywords:** African medicinal plants, chemical fingerprint, ethnopharmacology, herbal medicine, inflammation, *Lannea velutina* A.Rich., *Sorindeia juglandifolia* Planch. ex Oliv.

## Abstract

The dried leaves of *Lannea velutina* A.Rich. and *Sorindeia juglandifolia* Planch. ex Oliv. are commonly used in traditional medicine throughout West Africa to treat inflammatory diseases. The aim of the present study was to evaluate the anti-inflammatory activity of the standardized hydroethanolic (70%) extracts of these plants and to investigate the underlying mechanisms, with a focus on their antioxidant properties. The anti-inflammatory effects were evaluated using a rat model of induced paw edema, while the antioxidant activity was evaluated by DPPH^•^ radical scavenging and iron-reducing antioxidant power assays. Chemical fingerprint was achieved by LC-UV/DAD-ESI/MS and the main classes of secondary metabolites were quantified by colorimetric analysis. The results showed that *Sorindeia juglandifolia* extract significantly inhibited the increase in paw edema volume, with the maximum effect observed at doses of 100 and 200 mg/kg (20.51 ± 1.07% and 35.50 ± 6.90%, respectively). For *L. velutina*, the strongest inhibition was observed at 200 and 400 mg/kg (47.48 ± 11.37% and 35.40 ± 1.70%, respectively). Both extracts also showed remarkable antioxidant activity. Phenol derivatives were identified as the main classes of secondary metabolites, with *L. velutina* containing 350.1 ± 20.6 mg GAE/g DE and *S. juglandifolia* containing 463.4 ± 29.4 mg GAE/g DE. Ten phenolic markers were identified in *L. velutina* and six in *S. juglandifolia* extracts. The main components of *L. velutina* include myricetin-3-*O*-glucuronide, quercetin-3-*O*-glucuronide, catechin, and gallic acid, while *S. juglandifolia* contains gallic acid, isoquercitrin, and ethyl gallate. These results confirm the anti-inflammatory potential of *L. velutina* and *S. juglandifolia* and highlight their prospects as candidates for the development of standardized anti-inflammatory herbal medicines based on their chemical and biological properties.

## 1. Introduction

The use of medicinal plants has been a part of African culture for thousands of years. This knowledge has been passed down orally from generation to generation within specific population groups. These groups possess unique knowledge of the natural resources within their local areas and their application in treating diseases [1,2].

The World Health Organization’s traditional medicine strategy for 2014–2023 emphasizes the importance of traditional medicinal systems in Africa. Traditional healers can outnumber doctors by a ratio of 1:500 in some African countries. This type of medicine primarily relies on medicinal plants, but most of the plant species used are yet to be studied in ways that allow the establishment of quality and safety criteria and the understanding of the therapeutic activities for which they are used through the characterization of their mechanisms of action [3].

The anti-inflammatory activity exhibited by numerous medicinal plants, used in traditional medicinal systems for a long time, makes them an important focus for scientific research. Phenolic compounds are the most frequently identified natural products in these medicinal plants, and research has shown that antioxidant activity is consistently correlated to their presence in plant extracts, improving inflammatory symptoms through different mechanisms [4,5,6,7].

The *Anacardiaceae* family is among the largest groups of Angiosperms, comprising 80 genera and 967 species that occur in tropical and subtropical regions of hot or temperate climates. Several of these species are widely utilized by the local West African population as food and medicine [8].

*Lannea velutina* A. Rich. and *Sorindeia juglandifolia* Planch. ex Oliv. are examples of these species, whose different plant parts are sold in local markets as herbal medicines for treating various ailments [9,10].

*Lannea velutina* is a shrub or small tree that grows to 10 m in height and about 45 cm in diameter. The plant is characterized by imparipinnately compound leaves with opposite, entire leaflets. The flowers are tetramerous, with eight stamens, and exhibit sexual dimorphism; the anthers in female flowers are ovoid, reduced, and sterile. The ovary is four-locular, containing one apical pendulous ovule per locule, and is accompanied by three or four short styles with subglobose stigmas. In male flowers, the ovary is rudimentary. The fruit is a small, subreniform drupe that is compressed, with a thin mesocarp and a woody endocarp featuring an operculum.

*L. velutina* is native to western tropical Africa, existing from Senegal to Burkina Faso, as well as in Ghana, Chad, Central African Republic, Guinea Republic, and Guinea-Bissau. It dwells mainly in savannah woodland, in various types of soil, often in gravel or sandy places. In Guinea-Bissau, this species grows in woodland and savannah woodland, and its different plant parts are used for the treatment of inflammation, pain, muscle ailments, gastric ulcers, respiratory tract diseases, diarrhea, and fever [11,12]. The bark and leaf are used in a decoction serving as both a fortifying and detoxifying agent and the bark is also topically applied for treating ulcers, wounds, skin rashes, and cysts. Additionally, a mixture of root and leaf is used in cases of nephritis and rickets [13].

*Sorindeia juglandifolia* is a shrub or small tree distributed throughout western tropical and subtropical Africa, typically growing at altitudes ranging from 500 to 2000 m. It can be found in woodland and savannah woodland. The plant is a sarmentose shrub or small tree, reaching up to 9 m in height, and is generally glabrous. Leaves are compound, consisting of three to nine foliolate leaflets, with a petiole and rachis measuring 10–30 cm in length, and are subcylindrical, slightly compressed dorsally, and striate. The leaflets are subopposite to alternate and vary in shape from obovate to ovate, elliptic, or oblong-elliptic. Leaflets are large, with the terminal one reaching up to 22 × 11.5 cm, while lateral leaflets decrease in size toward the base. Flowers are whitish or yellowish with red tinges, with globose, obtuse buds.

*S. juglandifolia*’s fruits are edible and are used as a local food source [11]. In traditional medicine, the leaf is trampled, and the extract, obtained by decoction, is used to heal wounds and abscesses. The leaf is also used as a laxative and diuretic or to treat symptoms of sexual impotence [2,13], liver and kidney disease [14,15], and inflammatory conditions [16].

Our literature research showed that even though *L. velutina* leaf and *S. juglandifolia* leaf have been widely used in African traditional medicine to alleviate symptoms of inflammation and other diseases, no research has been conducted in order to substantiate this utilization [13].

Aqueous, methanolic, and hydroethanolic (80%) extracts from the bark and root bark of *L. velutina* demonstrated antioxidant activity in the 2,2-diphenyl-1-picrylhydrazyl (DPPH^•^) assay [12]. The bark ethanolic extract also showed in vitro antibacterial activity against Gram-negative (*Escherichia coli*, *Salmonella typhi*, *Shigella dysenteriae*) and Gram-positive bacteria (*Staphylococcus aureus* and *Bacillus cereus*) [12]. Methanolic extracts of different plant parts of *L. velutina* have previously shown in vitro antifungal activity against *Cladosporium cucumerinum* and *Candida albicans*; larvicidal activity against *Aedes aegypti*, *Anopheles gambiae* and *Culex quinque*-*fasciatus*; molluscicidal activity against *Biomphalaria glabrata*, *B. pfeifferi* and *Bulinus truncates*, and antioxidant activity [17].

The few studies conducted to evaluate the antibacterial properties of the methanolic extract of *S. juglandifolia* leaf have revealed its activity against five different bacterial strains (*Bacillus cereus*, *Escherichia coli*, *Enterococcus faecalis*, *Pseudomonas aeruginosa,* and *Staphylococcus aureus*) using the agar diffusion method [18,19]. Additionally, administering the aqueous extract of *S. juglandifolia* leaf orally to rats showed a protective effect against methotrexate-induced damage in the liver and kidneys [15].

In *L. velutina* root bark, phenolic compounds, namely dimeric, trimeric, and tetrameric proanthocyanidins (A- and B-Type) with a catechin starter unit and epicatechin extender units and epiafzelechin, and their methylated and galloylated derivatives have been identified [13,20].

Compounds belonging to different classes of secondary metabolites such as flavonoids, benzoic acids, tannins, terpenoids, and phenols have previously been identified in the leaf, fruit, and branch of *S. juglandifolia*, namely gallic acid, catechin, 2,3,6-trihydroxybenzoic acid, trihydroxymethyl-2,3,6-benzoate, 2″,6″-di-*O*-acetyl-7-*O*-methyl vitexin, 2″-*O*-acetyl-7-*O*-methyl vitexin, mearnsitrin, robustaflavone, 3-*O*-galloylcatechin, tachioside, 3-*β*-*O*-D-glucopyranosyl-*β*-stigmasterol, and methyl gallate [14,21,22]. 

Moreover, results from studies on the leaves hydroethanolic extract of *L. velutina* leaf, previously conducted by our team, showed no in vivo toxicity in animal models or in vitro genotoxicity. Briefly, in vivo oral repeated-dose toxicity was assessed in animals treated with *S. juglandifolia* extract at doses of 50, 400, and 1000 mg/kg body weight. Key parameters such as clinical signs, body weight changes, food consumption, mortality, and blood biochemical markers were monitored. The results indicated no significant alterations in general body weight or food intake. Although minor, non-critical changes in blood biochemistry were observed, they were not deemed of clinical significance, suggesting the extract’s safety at the tested doses. In vitro genotoxicity was assessed using the bacterial reverse mutation assay (Ame’s test), conducted with and without metabolic activation, in line with OECD guidelines. The results showed no genotoxic effects from either *S. juglandifolia* or *L. velutina* extracts at concentrations up to 5 mg/plate. The findings from both the toxicity and genotoxicity evaluations indicate that the hydroethanolic leaf extracts of *S. juglandifolia* and *L. velutina* are safe for medicinal use, as no significant adverse effects or genotoxic potential were observed. These studies also identified gallic acid and anacardic acid among the main marker compounds [23].

Our literature research showed that even though *L. velutina* leaf and *S. juglandifolia* leaf have been used in African traditional medicine to alleviate symptoms of inflammatory diseases, there is no scientific evidence that directly supports their use as anti-inflammatory agents [13]. Conducted studies so far have shown that the utilization of *L. velutina* in traditional medicine for the treatment of wounds, diarrhea, and fever can be related to the exhibited antibacterial in vitro activity, while its use in pain management, respiratory diseases, and inflammatory symptoms can be indirectly related to its antioxidant activity. As for *S. juglandifolia*, traditional medicine uses of the plant in wounds and abscesses can be related to the exhibited antibacterial in vitro activity. To contribute to the scientific validation of the traditional medicinal use of *L. velutina* and *S. juglandifolia* leaves, the present study aims to evaluate the potential in vivo anti-inflammatory and in vitro antioxidant activities of 70% hydroethanolic extracts, quantify the major chemical classes of secondary metabolites, and deeply investigate the principal marker secondary metabolites of each extract.

## 2. Results

### 2.1. Extracts Preparation

The extracts were prepared by maceration using 70% ethanol at room temperature with agitation, following the method previously described [23].

According to the obtained results shown in Table 1, *L. velutina* leaf yielded a higher amount of 70% hydroethanolic extract in comparison to *S. juglandifolia*.

### 2.2. Quantification of the Main Classes of Constituents Identified

Table 2 presents the results of the quantification of the main classes of secondary metabolites, such as total phenols, flavonoids, and hydrolysable and condensed tannins content, in 70% hydroethanolic extracts of *L. velutina* and *S. juglandifolia* leaves.

These contents were calculated and expressed as equivalent to the standards used in each assay.

The results showed that *L. velutina* leaf extract contained higher amounts of total condensed tannins, followed by hydrolysable tannins and flavonoids. As for *S. juglandifolia*, the primary phenolic components were predominantly hydrolysable tannins, followed by condensed tannins and flavonoids.

### 2.3. LC-UV/DAD-ESI/MS Analysis

**Figure 1:**
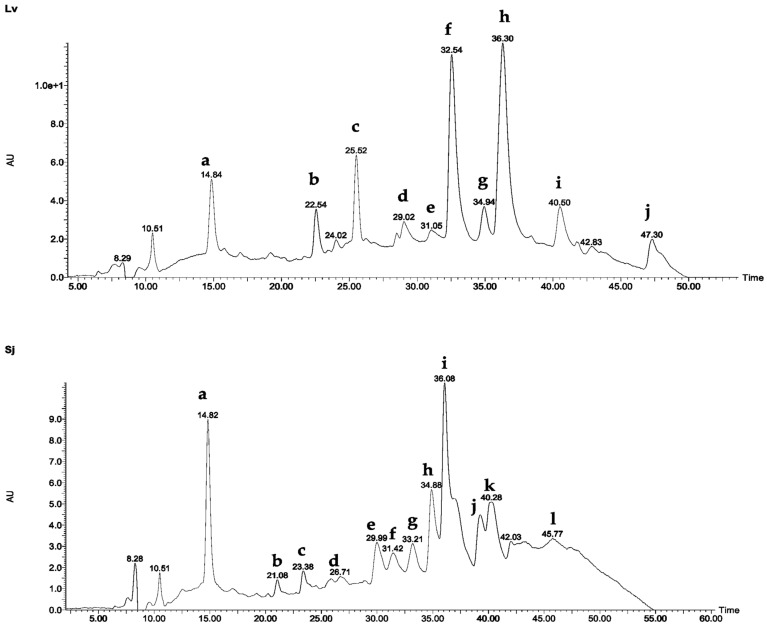
LC/MS chromatographic profiles of 70% hydroethanolic extracts of *L. velutina* (Lv) and *S. juglandifolia* (Sj) leaves.

The results of the *L. velutina* leaf 70% hydroethanolic extract (Lv), analyzed by LC-UV/DAD-ESI/MS, are shown in Figure 1 and summarized in Table 3. Similarly, the results for the *S. juglandifolia* leaf hydroethanolic extract (Sj) are illustrated in Figure 1 and detailed in Table 4.

The main compounds were tentatively identified through co-chromatography with authentic standards, comparison of their UV spectra, and mass spectrometric data, relying on the PubChem database and various scientific literature sources. Negative ionization data were utilized for identification purposes.

The obtained chromatograms for *L. velutina* leaf extract (Lv) showed a total of 10 major peaks (Figure 1, Table 3).

Peak **a** showed an [M − H]^−^ ion at *m*/*z* 169, a fragment ion at *m*/*z* 125 (possibly due to the loss of a CO_2_ molecule), and another fragment ion at *m*/*z* 97, possibly related to the rearrangement and loss of parts of the molecule, indicative of the compound’s structure. Based on the UV, fragmentation pattern, and co-chromatography with the standard, this compound was assigned as gallic acid [24].

Peak **b** showed an [M − H]^−^ ion at *m*/*z* 577 and fragment ions at *m*/*z* 425, 289, 245, 161, and 125, one of the most common fragmentation pathways of B-type proanthocyanidins and is suggestive of multiple fragmentation pathways, possibly due to the breaking of inter-flavan bonds, loss of galloyl groups, or ring opening. The complexity and variety of ions are indicative of the compound’s dimeric nature. A fragment ion at *m*/*z* 287 is a monomeric catechin unit formed due to quinone methide cleavage. Based on the UV spectra and fragmentation pattern, this compound was tentatively identified as type B dimeric procyanidin (dimers of (epi)catechin) [25].

Peak **c** exhibited an [M − H]^−^ ion at *m*/*z* 289 and fragment ions at *m*/*z* 245, 203, and 151, suggestive of typical fragmentation patterns of flavonoids, including ring fission and loss of specific groups like OH or CH_3_. Based on the UV, fragmentation pattern, and co-chromatography with the standard, this compound was assigned as catechin [24].

Peak **d**, like peak **b**, exhibited complex fragmentation typical of procyanidins, including losses specific to galloylation. Based on the UV and fragmentation pattern, this compound was assigned as type B dimeric procyanidin gallate [26].

Peak **e** exhibited an [M − H]^−^ ion at *m*/*z* 577, and the presence of ions like *m*/*z* 493, 431, and 363 suggests fragmentation pathways that include the loss of galloyl groups and other rearrangements, indicative of galloylation and a dimeric structure. By comparison of its fragmentation behavior with the previous literature, this peak was tentatively identified as type B dimeric procyanidin galloyl [25].

Peak **f** exhibited an [M − H]^−^ ion at *m*/*z* 493 and fragment ions like *m*/*z* 317 and 179, which are indicative of glucuronide conjugates and the typical fragmentation of myricetin by losing parts of the glucuronide moiety. This compound was identified as myricetin 3-*O*-glucuronide based on its spectral data, co-chromatography with the authentic standard, and a comparison to literature data [27].

Peak **g** exhibited an [M − H]^−^ ion at *m*/*z* 197, and fragment ions such as *m*/*z* 182 and 167 which suggest typical fragmentation patterns like demethylation, and fragment ion *m*/*z* 153 related to the loss carboxyl group. This compound was identified as gallic acid 3-5-dimethyl ether (syringic acid) based on its spectral data, co-chromatography with the authentic standard, and a comparison to literature data [28].

Peak **h** exhibited an [M − H]^−^ ion at *m*/*z* 477, and fragment ions *m*/*z* 301, 169, and 151, indicating the presence of quercetin aglycone formed by the loss of the glucuronic acid moiety and subsequent fragmentation. Based on the UV and fragmentation patterns previously reported in published literature, this compound was identified as quercetin 3-*O*-glucuronide [29].

Peak **i** exhibited an [M − H]^−^ ion at *m*/*z* 447, and fragment ions *m*/*z* 469, 301, 269, and 146, suggesting typical fragmentation patterns for quercetin derivatives due to the loss of a rhamnosyl moiety. This compound was identified as quercitrin (quercetin-3-*O*-rhamnoside) based on LC/UV-DAD co-chromatography with the authentic standard and a comparison to literature data [30].

Peak **j** exhibited an [M − H]^−^ ion at *m*/*z* 491, and the presence of ions *m*/*z* 447, 315, and 301, which indicates glucuronide conjugation with a methyl group and is suggestive of typical fragmentation of methylated quercetin derivatives. This compound was identified as 3-methyl-quercetin-3-*O*-*β*-*D*-glucoronide (isorhamnetin 3-*O*-glucuronide) based on the UV and fragmentation patterns previously reported in the published literature [31].

The obtained chromatograms for *S. juglandifolia* leaf extract (Sj) also showed a total of six major peaks as shown in Figure 1 and Table 3.

Similar to Lv extract, peak **a** showed a [M − H]^−^ ion at *m*/*z* 169 and fragment ions at *m*/*z* 125, consistent with the loss of CO_2,_ and *m*/*z* 97, formed during further breakdown. Based on the UV, fragmentation pattern, and co-chromatography with the standard, this compound was also identified as gallic acid [24].

Peak **d** showed a [M − H]^−^ ion at *m*/*z* 289 and fragment ions at *m*/*z* 245 (loss of CO_2_), 137, 125, and 109 suggesting a retro-Diels–Alder reaction (RDA). The *m*/*z* 137 and 125 are indicative of the cleavage of the C-ring in flavan-3-ols, which is a common fragmentation pathway for catechins. Based on the UV, fragmentation pattern, and co-chromatography with the standard, this compound was identified as (−)-epicatechin [24,32].

Peak **e** showed a [M − H]^−^ ion at *m*/*z* 457 and fragments like the *m*/*z* 305 ion, corresponding to [M − H]^−^ of epigallocatechin (EGC), and *m*/*z* 288, corresponding to the loss of gallate moiety. A further breakdown due to deprotonated and decarboxylated ions of the gallic acid moieties resulted in ions at *m*/*z* 169 and *m*/*z* 125. Based on the UV, fragmentation pattern, and co-chromatography with the standard, this compound was identified as epigallocatechin gallate (EGCG) [33,34].

Peak **h** showed a [M − H]^−^ ion at *m*/*z* 197, representing the mass of the deprotonated molecule, and a fragment ion at *m*/*z* 169, indicating demethylation to gallic acid. Based on the UV, fragmentation pattern, and co-chromatography with the standard, this compound was identified as ethyl gallate [35].

Peak **i** showed a [M − H]^−^ ion at *m*/*z* 463 and fragmentations ions at *m*/*z* 301 and *m*/*z* 179, indicating a loss of a sugar moiety and the aglycone part of the molecule (characteristic of quercetin glycosides). Based on the UV, fragmentation pattern, and co-chromatography with the standard, this compound was identified as isoquercitrin [36].

Similar to the compound **i** in *L. velutina*, peak **k** showed a [M − H]^−^ ion at 447 *m*/*z* with fragmentation ions at *m*/*z* 301, 269, and 146, indicating the typical fragmentation patterns for quercetin derivatives, the cleavage of the sugar moiety (loss of the rhamnosyl moiety), and further fragmentation of the aglycone. Based on the UV, fragmentation pattern, and co-chromatography with the standard, this compound was identified as quercitrin [30].

A comparative analysis of the retention times and ultraviolet photodiode array (HLPC-UV/DAD) spectra of standards with the obtained peaks of the respective chromatograms showed the presence of gallic acid and quercitrin in both *S. juglandifolia* (Sj) and *L. velutina* (Lv) 70% hydroethanolic extracts (Figure 1). The identification of these compounds was also confirmed using co-chromatography data. Gallic acid (**a**) and isoquercitrin (**i**) were detected as the major marker compound of *S. juglandifolia*, and myricetin 3-*O*-glucuronide (**f**) and quercetin 3-*O*-glucuronide (**h**) were detected as the major marker compound of *L. velutina* extracts. Despite our efforts, compounds **b**, **c**, **f**, **g**, **j,** and **l** were not identified. Further studies should be conducted to complete the phytochemical characterization of *S. juglandifolia*.

### 2.4. Anti-Inflammatory Activity

**Figure 2:**
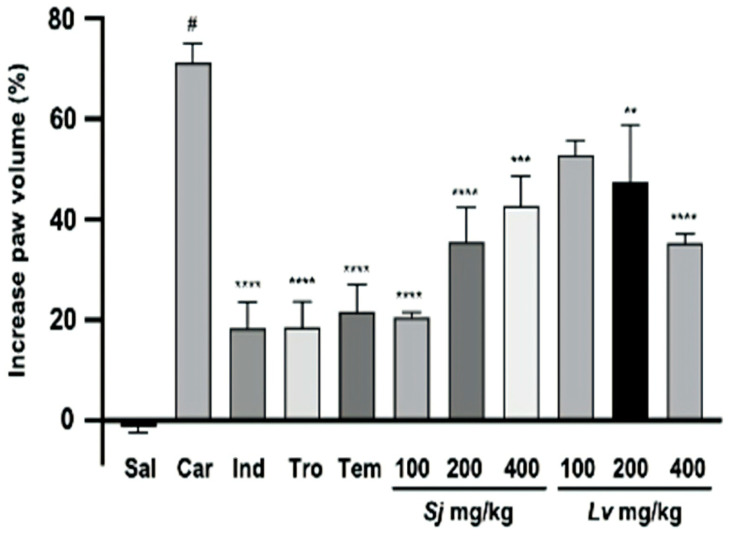
Anti-inflammatory activity of 70% hydroethanolic extracts of *L. velutina* and *S. juglandifolia* leaves by inhibition of carrageenan-λ-induced paw edema (1%) in male Wistar rats. The data are displayed as averages and their default errors. **Abbreviations**: Sal—saline; Car—carrageenan; Ind—indomethacin; Tro—Trolox; Tem—Tempol; *S. juglandifolia*; *L. velutina* **** *p* < 0.0001; *** *p* < 0.001; ** *p* < 0.01; versus carrageenan group; # *p* < 0.0001 versus saline (control) group.

The results from the administration of *L. velutina* and *S. juglandifolia* leaf extracts at concentrations of 100, 200, and 400 mg/kg, as well as reference substances with anti-inflammatory and antioxidant activities (indomethacin, Trolox, and Tempol), are presented in Figure 2. These results were expressed as a percentage of inhibition of the rat paw edema increase induced by carrageenan-λ (1%) and were statistically significant in all analyses performed.

An analysis of the results showed that 6 h after induction of edema, the maximum inhibition of paw volume increase was found at doses of 100 and 200 mg/kg (20.51 ± 1.07% and 35.50 ± 6.90%) in animals treated with the *S. juglandifolia* leaf extract and at doses of 200 and 400 mg/kg (47.48 ± 11.37% and 35.40 ± 1.70%) in animals treated with *L. velutina* leaf extract. These results demonstrate that there may be a dose-dependent relation between the inhibition of the animal’s paw volume increase and the administered extracts.

A statistically significant difference (*p* < 0.0001) was observed between the carrageenan group and the negative control group, which demonstrates the validity of the experimental model of inflammation induction. Significant statistical differences were observed between the carrageenan group and the positive control groups, indomethacin, Trolox, and Tempol (*p* < 0.0001); groups treated with *S. juglandifolia* 100 mg/kg and 200 mg/kg (*p* < 0.0001) and *S. juglandifolia* 400 mg/kg (*p* < 0.001); and groups treated with *L. velutina* 200 mg/kg (*p* < 0.01) and 400 mg/kg (*p* < 0.0001).

We found no statistically significant differences (*p* < 0.05) between the negative control group and the indomethacin, Trolox, and *S. juglandifolia* 100 mg/kg groups, which allowed us to infer that the paw edema increase significantly prevented the administration of these treatments.

The results obtained in this study indicate that the hydroethanolic extracts of *S. juglandifolia* leaf at doses of 100, 200, and 400 mg/kg and *L. velutina* leaf at doses of 200 and 400 mg/kg significantly reduced the paw edema induction of the Wistar rats when compared to the carrageenan group.

### 2.5. Antioxidant Activity

Table 5 summarizes the results related to the antioxidant activity of *L. velutina* and *S. juglandifolia* leaves obtained in the DPPH^•^ and FRAP assays.

Regarding the DPPH^•^ assay, we found that *L. velutina* leaf extract was the one with the highest antioxidant activity (IC_50_ = 1253.36 μg/mL). The lowest antioxidant activity was observed for *S. juglandifolia* leaf extract (IC_50_ = 1517.86 μg/mL). Both formulations had lower antioxidant activity than the ascorbic acid positive control (IC_50_ = 48.3 μg/mL).

## 3. Discussion

The discovery of drugs from plant origins with a long-standing history of use holds promise for developing effective treatments for inflammation-related conditions. Moreover, using plants as raw materials for drug discovery is often more sustainable and accessible, especially for developing countries, leading to more affordable treatments that are easier to produce and distribute [37].

*Lannea velutina* and *Sorindeia juglandifolia* are among such promising plants with potential medical applications due to their unique properties and historical use in African traditional medicine for the treatment of symptoms of conditions caused by oxidative stress damage, such as injuries, inflammation, and pain [14].

Based on the results of this study, *L. velutina* leaf yielded a higher amount of 70% hydroethanolic extract compared to *S. juglandifolia*. However, the quantification of the main secondary metabolites and a more detailed analysis of their LC/MS chromatograms indicated higher amounts of phenolic derivatives in *S. juglandifolia* compared to *L. velutina*.

The presence of flavonoids (quercetin derivatives), hydrolysable (gallic acid derivatives), and condensed tannins (catechin derivatives) was evident in both species.

The analysis of Table 2 and Table 5 confirmed a strong correlation (r = −0.96895) between antioxidant activity and the total phenol content. This coefficient’s approach to −1 indicates a clear inverse relationship where a higher content of these compounds correlates with higher antioxidant power (lower IC_50_).

Both *S. juglandifolia* and *L. velutina* leaf hydroethanolic extracts (70%) have shown considerable antioxidant (DPPH^•^ radical scavenging activity) and anti-inflammatory (inhibition of carrageenan-induced paw edema) potential, exhibiting a concentration-dependent effect in both assays. The dose-dependent anti-inflammatory activity of a certain compound/extract is a well-documented phenomenon, where higher doses typically correlate with greater suppression of inflammatory responses [38,39,40,41]. However, our experimental findings with the *S. juglandifolia* extract have revealed a reversed pattern of expectation.

The obtained inverse dose-dependent effect on carrageenan-induced paw edema by *S. juglandifolia* extract revealed that low to moderate doses demonstrated a significant reduction in inflammation, whereas, in higher doses, the inflammation and edema formation unexpectedly persisted. This paradoxical outcome may indicate an issue with extract preparations, possibly involving the precipitation of significant amounts of pharmacologically active compounds during the assay or the presence of antagonistic components that enhance the inflammatory process and lead to increased edema. Thus, at lower doses of *S. juglandifolia* extract, this antagonistic effect may be negligible, and the anti-inflammatory effect can be observed. Furthermore, it could reflect the complex interplay between dosage, cellular pathways, and immune responses, which requires further investigation and highlights the importance of comprehensive dose–response studies to optimize therapeutic strategies effectively [42].

As of now, no data concerning the anti-inflammatory activity of either species or the antioxidant activity of *S. juglandifolia* have been reported. *L. velutina* extracts, however, have shown in vitro antioxidant activity as indicated by several studies [9,12,20,43]. In a recent study by Kaboré et al., 2023 [44], the leaf methanol extract exhibited the highest DPPH^•^ radical scavenging activity, with an IC_50_ of 15.38 µg/mL, compared to hexane, ethyl acetate, dichloromethane, and aqueous extracts (IC_50_: 546.56 µg/mL, 51.64 µg/mL, 409.36 µg/mL and 101.40 µg/mL, respectively). The results obtained in our study are consistent with these findings, emphasizing the critical role of solvent selection in the extraction process and subsequent activities.

Phenolic compounds are key players in antioxidant mechanisms, particularly in scavenging free radicals and reducing oxidative damage [4,5].

Our data shows that *Lannea velutina* and *Sorindeia juglandifolia* extracts have both anti-inflammatory and antioxidant properties. The presence of phenolics such as catechin, quercetin, and gallic acid derivatives in both *L. velutina* and *S. juglandifolia* is aligned with the observed antioxidant activity after the administration of both extracts. The chemical class of phenolic compounds is well known for the capacity to scavenge free radicals and inhibit oxidative stress, which is a major factor in chronic inflammation [4].

Phenolic compounds, particularly flavonoids like quercetin and catechins, have also been shown to modulate inflammatory pathways by inhibiting the production of pro-inflammatory cytokines and reducing the activation of NF-κB, a key factor in the inflammatory response [4]. This aligns with our obtained findings using the rat paw edema model, where inhibition rates were observed with both extracts.

Given these facts, as phenolic compounds were the primary components of these extracts, it is likely that they are responsible for reducing paw edema in rats [45,46].

## 4. Materials and Methods

### 4.1. Harvesting, Drying, and Identification of Plant Material

The plant material was composed of leaves and was harvested in October 2015 in Bissora (12°13′23″ N, 15°26′51″ W), Guinea-Bissau. Identification was performed by Professor Luís Catarino of the Department of Plant Biology, Faculty of Sciences, Universidade de Lisboa through comparison of the morphological features of the samples with voucher specimens available at the National Museum of Science & Natural History (LISC) of the Universidade de Lisboa based on species’ description in Flora of West Tropical Africa. The voucher specimens LC2131 (*Lannea velutina*) and LC2129 (*Sorindeia juglandifolia*) were deposited in the herbarium of LISC. The plant material was air-dried in the shade and transported to the pharmacognosy laboratory of the Faculty of Pharmacy, Universidade de Lisboa, where the analyses were carried out. These activities had previously been conducted by our team.

### 4.2. Reagents

Acetone (≥99.5%), aluminum chloride (98%), ascorbic acid (99%) (A1300000), gallic acid (98%) (PHL89198), carrageenan, (±)-catechin (≥98%) (PHL89172), DPPH^•^ (2,2-diphenyl-1-picrylhydrazyl) (≥90%), (−)-epicatechin (≥98%) (PHL89192), (−)-epigallocatechin gallate (≥95%) (PHR1333), ethyl gallate (≥95%) (PHL83080), indomethacin (98.5–100.5%), Tempol (≥97%), Trolox (≥98%), and 2,4,6-tris(2-pyridyl)-s-triazine (TPTZ), (≥99.0%) were purchased from Sigma-Aldrich^®^ (St. Louis, MO, USA).

Ferric (Iron III) chloride hexahydrate (≥99%), hydrochloric acid (37%), potassium iodate (99.5%), sodium acetate trihydrate (≥99%), sodium carbonate (≥99.5%), and sodium hydroxide and sodium nitrite (98.0%), were obtained from Merk (Darmstadt, Germany).

Acetonitrile was purchased from Honeywell Riedel-de HaënTM (Seelze, Germany). Ferrous (Iron II) sulfate heptahydrate (>98%) was purchased from May and Baker laboratory chemicals (Dagenham, England), Folin–Ciocalteu was purchased from Biochem chemopharma (Cosne-Cours-sur-Loire, France). Methanol (≥99.8%) and *n*-Butanol (99.5%) from Fisher Chemicals^®^ (Leicestershire, UK) and sodium chloride (0.9%) from B. Braun Medical Lda (Oeiras, Portugal) were acquired. In preparing all solutions and dilutions, ultra-pure water from a Milli-Q water purification system, Millipore (Molsheim, France), was used.

### 4.3. Preparation of the Hydroethanolic Extracts

The dried plant leaves were grinded and extracted until exhaustion with ethanol 70% at room temperature under agitation. *L. velutina* was extracted in a plant–solvent ratio of 1:23, and *S. juglandifolia* 1:27. Separation of the solution was performed by vacuum filtration, using a Yamato WP-25 water pump coupled to a G4 glass plate filter and a kitasate. The obtained extracts were concentrated to a dry residue, using a Buchi rotavapor model R 210, coupled to a membrane pump, type A-39 Brand Wheaton^®^, at a temperature below 40 °C and freeze-dried.

### 4.4. Quantification of the Main Classes of Constituents Identified

#### 4.4.1. Total Phenol Quantification

Total phenolic quantification of the two hydroethanolic extracts, was performed using Folin–Ciocalteu method [47] with modifications.

Briefly, after preparation of the extract dilutions (0–1000 μg/mL), 0.5 mL of extracts, 2 mL of Folin–Ciocalteu Reagent (10%), and 1.6 mL of sodium carbonate (75 g/L) were added to each test tube. After 2 h of incubation in dark at room temperature, the absorbance was determined using a Hitachi U-2000 spectrophotometer (Tokyo, Japan) at a wavelength of 760 nm, using distilled water as control. Gallic acid (0–100 μg/mL) was used to elaborate the standard curve (y = 0.0097x + 0.0701) and the content in total phenols was expressed in milligrams of gallic acid equivalents (GAEs) per gram of the leaves ethanolic extracts of *L. velutina* and *S. juglandifolia*.

#### 4.4.2. Total Flavonoid Quantification

According to the method described by Olivera et al., 2008 [48], concentrations of (0–2000 μg/mL) of each extract were prepared. To 0.5 mL of extracts, 2 mL of distilled water and 150 µL of NaNO_2_ (5%) were added to each tube, incubated for 5 min, followed by addition of 150 µL of AlCl_3_ (10%), and incubated for another 6 min. Then, 1 mL of NaOH 1M was added and tubes were incubated for 20 min at 18 °C under the light. Absorbance was determined using a Hitachi U-2000 spectrophotometer (Tokyo, Japan) at 510 nm. Catechin (0–100 μg/mL) dissolved in distilled water was used to elaborate the standard curve (y = 0.005x + 0.004) and the total flavonoid content expressed in milligrams of catechin equivalents (CAEs) per gram of the leaves ethanolic extracts of *L. velutina* and *S. juglandifolia*.

#### 4.4.3. Quantification of Condensed Tannins

Condensed tannins were quantified using methodologies described by Wilfred Vermerris, 2006 [49], with modifications. The assay was performed by adding 0.5 mL of plant extract (dilutions of 0–2000 μg/mL), followed by 5 mL of an acidic solution (HCl/n-butanol, 2:3). The samples were then placed in a water bath at 95 °C for 15 min. Absorption was determined using a Hitachi U-2000 spectrophotometer (Tokyo, Japan) at 530 nm. Catechin (0–200 μg/mL) was used to elaborate the standard curve (y = 0.0002x + 0.0324) and the content in condensed tannins was expressed in milligrams of CAEs per gram of the ethanolic extracts of *L. velutina* and *S. juglandifolia* leaves.

#### 4.4.4. Quantification of Hydrolysable Tannins

Hydrolysable tannins were quantified by adding 1.5 mL of a saturated potassium iodate solution to 3.5 mL of the extracts (1 mL of sample and 2.5 of 20% acetone/water), followed by a 40 min incubation at 0 °C (according to the methods previously described, with modifications [49,50]. Absorption was determined using a Hitachi U-2000 spectrophotometer (Tokyo, Japan) at 550 nm.

Gallic acid (0–600 μg/mL) was used to elaborate the standard curve (y = 0.001x + 0.054) and the content in hydrolyzable tannins was expressed in milligrams of GAEs per gram of the leaves ethanolic extracts of *L. velutina* and *S. juglandifolia*.

### 4.5. HPLC-UV/DAD-MS/ESI Phytochemical Profile

Samples were solubilized in acetonitrile (5 mg/mL) and filtered through a polytetrafluoroethylene (PTFE) syringe filter (0.2 μm) before the analysis. The samples were then injected in a volume of 10 μL into a LiChrospher^®^ 100 RP-18 end-capped particle size 5 μm, 100 Å, LiChroCART^®^ 250 × 4 mm (Merck, Darmstadt, Germany) and separated by HPLC Waters Alliance 2695 coupled to a Waters 996 photodiode array detector (PDA) (Waters Corporation, Milford, MA, USA).

Phytochemical profile was established according to the method previously described by us with some modifications [23]. Two mobile phases were employed: water + 0.1% formic acid (solvent A) and acetonitrile (solvent B). Chromatographic separation was performed at a flow rate of 0.3 mL/min using the following gradient elution program: from 0 to 20 min → 95:5 (A:B) to 80:20 (A:B) and from 20 to 60 min → 80:20 (A:B) to 50:50 (A:B). This was followed by washing and reconditioning of the column. The temperature of the column thermostat was 25 °C. Data were registered and analyzed using Waters Millennium^®^32 Chromatography Manager (Waters Corporation, Milford, MA, USA). The detection was performed on PDA detector in a wavelength range of 210–700 nm.

The MS/ESI analyses and mass spectroscopy were performed using a triple quadrupole Mass Spectrometer (Micromass^®^ Quatro MicroTM API, Waters^®^, Drinagh, Ireland) The ionization of the compounds was carried out by an electrospray source in negative mode (ESI-) at 20 V cone voltages. For data processing, the Waters MassLynx™ Software version 4.1 (Drinagh, Ireland) was used.

### 4.6. Anti-Inflammatory Activity

Anti-inflammatory activity was evaluated using the carrageenan-induced paw edema protocol in rats, described by Rocha et al., 2015 [51]. This experimental protocol was approved in February 2016 by the Ethics Committee for Animal Experiments (CEEA) of the Faculty of Pharmacy of the Universidade de Lisboa (protocol CEEE-002/16). The experiments were performed in agreement with European and Portuguese ethical requirements [52,53].

#### 4.6.1. Animals

Male Wistar rats (154–309 g) were purchased from Harlan Laboratories Inc. (Barcelona, Spain). Animals were then distributed in cages with a number of six rats per group in the bioterium facility of the Faculty of Pharmacy of the Universidade de Lisboa; the rats were subject to a controlled temperature (22 ± 2 °C), humidity (60–80%), and luminosity (light/dark cycle of 12 h) and had access to water and rat chow ad libitum for three weeks before the start of the study.

#### 4.6.2. Experimental Protocol

Sixty-seven male Wistar rats were randomized by their weight and divided into eleven groups. Drug dosage and administration were based on studies with plants of the same genus and family [33]. Administration of the drugs, aqueous extracts, or controls was performed via oral gavage of 10 mL/kg body weight (BW) [54]. Moreover, the three doses (100, 200, and 400) were selected and administered, respecting the maximum limit of 1000 mg/kg/day in rodents and non-rodents. Group 1 (negative control): animals received distilled water (*n* = 5); Group 2 (vehicle control group—carrageenan-λ): animals received distilled water (*n* = 11); Group 3 (indomethacin group): animals pre-treated with indomethacin (10 mg/kg) (*n* = 5); Group 4 (Trolox group): animals pre-treated with Trolox (30 mg/kg) (*n* = 5); Group 5 (Tempol group): animals pre-treated with Tempol (30 mg/kg) (*n* = 5); Groups 6–8 (Sj groups): animals pre-treated with *S. juglandifolia* extracts at 100, 200, and 400 mg/kg (*n* = 6 for each dose); Groups 9–11 (Lv groups): animals pre-treated with *L. velutina* extract at 100, 200, and 400 mg/kg (*n* = 6 for each dose).

After one hour, paw edema was induced in all rats by a single subplantar injection of 0.1 mL of 1% carrageenan saline solution in the left hind paw (except in the negative control group that received a subplantar injection of 0.1 mL of sterile saline).

Paw volume was measured immediately after the injection of carrageenan (V0) and six hours later (V6) using a Digital Plethysmometer LE7500 (LSI Letica Scientific Instruments, Porto, Portugal).

Percentage inhibition of carrageenan induced paw edema was calculated according to the following equation: % = [(V6 − V0)/V0] × 100, and the obtained results were expressed as the percentage of paw volume increase compared to the initial volume.

### 4.7. Determination of Antioxidant Activity

The in vitro antioxidant activities of 70% hydroethanolic extracts of both *L. velutina* and *S. juglandifolia* were evaluated using DPPH^•^ and FRAP colorimetric assays.

All values were determined in 3 sets of experiments and evaluated in triplicate using a Hitachi U-2000 UV-Vis spectrophotometer (Tokyo, Japan). The results are presented as mean ± SD. DPPH^•^ assay and the FRAP assay [55]. All the tests were performed in triplicate.

#### 4.7.1. DPPH^•^ Assay

The free radical scavenging activity was determined by the previously described DPPH^•^ method [56,57]. DPPH^•^ solution (3.9 mL, 6 × 10^−5^ M in methanol) was mixed with 100 µL of diluted extract/standard solution/blank(water). After 30 min of incubation at room temperature, the absorbance of samples and standard solution were measured at 517 nm.

Results were presented in inhibitory concentration (IC_50_ value), which represents the concentration of sample required to scavenge 50% of DPPH^•^ free radicals. The percentage of DPPH^•^ free radical scavenging activity was calculated using the following formula: % scavenging = [Absorbance of control − Absorbance of test sample/Absorbance of control] × 100. The obtained results were then compared to the equation obtained from the ascorbic acid linear standard curve (25–200 µg/mL): y = 0.616x − 1.1702, R^2^ = 0.9989, using Microsoft Excel Software Program.

#### 4.7.2. Ferric Reducing Antioxidant Power Assay

In order to quantify the antioxidant potential within both *L. velutina and S. juglandifolia* leaf extracts, the ferric reducing antioxidant power (FRAP) assay was *performed* according to the method described by Benzie and Strain (1996) with some modifications [55].

To conduct the assay, 3 mL of freshly prepared FRAP reagent (consisting of 300 mM acetate buffer pH 3.6, 10 mM TPTZ in 40 mM HCl, 20 mM FeCl_3_, and 6H_2_O in the ratio of 10:1:1 at the time of use) was combined with 100 µL of plant extracts (1000–5000 μg) placed in the water bath at 37 °C. The absorbance was then measured at 593 nm after 4 min using a Hitachi U-2000 spectrophotometer (Tokyo, Japan). Ascorbic acid concentrations (25–175 µg/mL) were used to obtain a standard curve with an equation of y = 0.616x − 1.1702, R^2^ = 0.9989, and the results were expressed as mmol or µg ascorbic acid equivalents/g dry extract (DE).

### 4.8. Statistical Analysis

Microsoft Excel version 16.49 (Microsoft Corporation, Redmond, WA, USA) and Graphpad Prisma^®^ software version 10.4.1 (GraphPad Software Inc., San Diego, CA, USA) were used, and a simple variance analysis (one-way ANOVA) and Donnett’s Multiple Comparison Test were performed.

## 5. Conclusions

Although the role of medicinal plants in treating various ailments within these communities is crucial, scientifically validating their traditional uses enhances their safety and efficacy. Our findings contribute to this scientific validation of the use of traditional formulations based on *L. velutina* and *S. juglandifolia,* reinforcing the evidence that these plant extracts have considerable content of phenolic compounds, which are directly related to the exhibited anti-inflammatory and antioxidant activities. *S. juglandifolia* leaf hydroethanolic extract exhibited the highest content of phenolic compounds and demonstrated strong anti-inflammatory activity, as administering a dose of 100 mg/kg produced similar results to those of the positive controls in the paw edema rat assay. Moreover, the activities exhibited by these two species of the *Anacardiaceae* family suggest significant potential for developing herbal medicines; however, further research is necessary to better understand their safety and efficacy as drugs.

## Figures and Tables

**Table 1:** Quantification of yield and drug-extract ratio in 70% hydroethanolic extracts of *L. velutina* and *S. juglandifolia* leaves.

Species	DLW (g)	DE (g)	DER	Yield
*L. velutina*	207.5	40.5	5.1:1	19.5%
*S. juglandifolia*	288.6	53.4	5.4:1	18.5%

Abbreviations: DE: dried extract; DER: drug extract ratio; DLW: dried leaves weight.

**Table 2:** Quantification of the major chemical classes of secondary metabolites in 70% hydroethanolic extracts of *L. velutina* and *S. juglandifolia* leaves.

Sample	TPC (mgGAE/gDE)	TFC (mgCAE/gDE)	TCTC (mgCAE/gDE)	THTC (mgGAE/gDE)
*L. velutina*	350.1 ± 20.6	152 ± 10.1	222 ± 12.5	170.8 ± 4.5
*S. juglandifolia*	463.4 ± 29.4	72.1 ± 2.8	139.3 ± 12	162.4 ± 1.3

Abbreviations: CAE: catechin equivalent; DE: dried extract; GAE: gallic acid equivalent; TCTC: total condensed tannin content; TFC: total flavonoid content; THTC: total hydrolysable tannin content; TPC: total phenolic content.

**Table 3:** LC-UV/DAD-ESI/MS identification of *L. velutina*’s major marker compounds.

Peak	*t*_R_ (min)	UV λ_max._ (nm)	[M − H]^−^ (*m*/*z*)	MS/MS Fragment Ions (*m*/*z*)	Assignment
a	14.84	272	169	125, 97	gallic acid
b	22.54	279	577	425, 289, 245, 161, 125	Type B dimeric procyanidin
c	25.52	279	289	245, 203, 151, 137, 125, 109	catechin
d	29.02	278	729	577, 431, 385, 289, 247, 245, 179	Type B dimeric procyanidin gallate
e	31.05	276	577	493, 431, 363, 341, 287, 203, 179	Type B dimeric procyanidin galloyl
f	32.54	250, 263sh, 300sh, 356	493	317, 179, 97	myricetin 3-*O*-glucuronide
g	34.94	272	197	182, 167, 153, 125	gallic acid 3-5-dimethyl ether (syringic acid)
h	36.30	258, 266sh, 294sh, 355	477	463, 301, 169, 151	quercetin 3-*O*-glucuronide
i	40.50	254, 269sh, 294sh, 348	447	469, 301, 269, 255, 146	quercitrin (quercetin-3-*O*-rhamnoside)
j	47.30	255, 269sh, 294sh, 354	491	447, 315, 301	3-methyl-quercetin-3-*O*-*β*-*D*-glucuronide

Abbreviations: *m*/*z*—mass-to-charge ratio; [M − H]^−^: negative mass electrospray ionization mode; *t*_R_: retention time; λ_max_: wavelength of maximum absorbance.

**Table 4:** LC-UV/DAD-ESI/MS identification of *S. juglandifolia* major marker compounds.

Peak	*t*_R_ (min)	UV λ_max._ (nm)	[M − H]^−^ (*m*/*z*)	MS/MS Fragment Ions (*m*/*z*)	Assignment
a	14.82	272	169	125, 97	gallic acid
d	26.71	275	289	245, 137, 125, 109	(−)-epicatechin
e	29.99	274	457	305, 288, 169,125, 97	epigallocatechin gallate
h	34.88	273	197	169	ethyl gallate
i	36.08	254, 266sh, 294sh, 347	463	431, 301, 179	isoquercitrin
k	40.28	255, 268sh, 294sh, 347	447	469, 301, 269, 255, 146	quercitrin

Abbreviations: *m*/*z*—mass-to-charge ratio; [M − H]^−^: negative mass electrospray ionization mode; *t*_R_: retention time; λ_max_: wavelength of maximum absorbance.

**Table 5:** Determination of the antioxidant activity of 70% hydroethanolic extracts of *L. velutina* and *S. juglandifolia* leaves.

Sample	DPPH^•^	FRAP	
	(IC_50_) μg/mL	µgAAE/mgDE	mmolAA/gDE
*L. velutina*	1253.4	159.3 ± 16.8	0.91
*S. juglandifolia*	1517.9	123.4 ± 11.3	0.72

Abbreviations: AA: ascorbic acid; AAE: ascorbic acid equivalent; DDPH: 2,2-diphenyl-1-picrylhydrazyl; DE: dried extract; FRAP: ferric reducing antioxidant power; IC_50_: half maximal inhibitory concentration.

## Data Availability

Data are contained within the article.
